# Impact of Biophysical Mechanisms on Urban Heat Island Associated with Climate Variation and Urban Morphology

**DOI:** 10.1038/s41598-019-55847-8

**Published:** 2019-12-20

**Authors:** Ressy Fitria, Daeun Kim, Jongjin Baik, Minha Choi

**Affiliations:** 1 Environment and Remote Sensing Laboratory, Department of Water Resources, Graduate School of Water Resources, Sungkyunkwan University, Suwon, 16419 Republic of Korea; 2 Center for Built Environment, The Built Environment Department, Sungkyunkwan University, Suwon, 16419 Republic of Korea

**Keywords:** Urban ecology, Environmental impact

## Abstract

The rapid development of urban areas can potentially alter hydro-meteorological fluxes and lead to the Urban Heat Island (UHI) phenomenon. In this study, UHI intensity and its driving factors were estimated using the Community Land Model (CLM) in cities of Tokyo, Phoenix, Bandung, and Quito, with different landscapes and climates, as a step in risk assessment of urbanization phenomena. The UHI magnitude increased along with the ratio of the height to width (H/W) of urban canyons in cities with the same latitude, especially during the daytime, when Quito (Tokyo) had a higher UHI than Bandung (Phoenix). El Niño-Southern Oscillation (ENSO) events, such as El Niño and La Niña, contributed to UHI variability, during which the cities in the western (eastern) part of Pacific Ocean experienced a higher UHI during El Niño (La Niña). The UHI differences from total biophysical drivers between these events were highest in Tokyo during the daytime as a result of convection process, and in Phoenix during the nighttime due to the hot arid climate of the city. Our results suggest the need to consider climate variation beyond local site characteristics when mitigating heat stress and making decisions regarding urban development.

## Introduction

Urbanization is a rapidly growing global trend, and the intensity of urban development is expected to increase in the future^1^. Urban growth leads to an increase in impervious surfaces that can influence the hydrological process and meteorological conditions of entire catchments^2,3^. Impervious surfaces affect the land surface temperature (LST) of urban areas^2^ due to low soil moisture and reduced evapotranspiration^4^. The higher LST of urban areas compared with surrounding rural areas is widely known as the urban heat island (UHI) phenomenon^5^ and has become a significant problem in recent years due to urbanization and industry activity^6,7^. This phenomenon affects rainfall patterns and quantity, which leads to depletion of water resources associated with low air moisture and atmospheric instability^4,8–10^.

For quantification of UHI and to perform further spatial analysis of UHI characteristics, research methods have expanded to include different sizes and characteristics of cities, such as a single city, megacity, and city agglomeration^11^. Early studies of UHI were based on meteorological observations by analyzing the difference in air temperature of urban and suburban areas^12^. This method only worked efficiently for a single point because observation stations were sparsely distributed^11^. To overcome the limitations of point-based UHI and to quantify regional-based UHI, remote sensing technology that offers wide coverage and good synchronization data has been recently developed^11,13,14^. However, many satellite data used for UHI estimations suffer from low temporal resolution, which limits the ability to estimate continuous UHI intensity.

Urban land surface models are among the methods used to estimate UHI intensity with a higher temporal resolution. Several urban models have attracted research attention, such as the Integrated Urban Model (IUM)^15^ developed from the Common Land Model (CoLM)^16^ and the Community Land Model Urban (CLMU), which had integrated the parameterization of urban surfaces coupled to the Community Land Model (CLM) since the fourth version^1^. These models produce a number of land surface variables from physical processes with sufficient complexity for urban parameterization^1^. The availability of these variables enables exploration of land cover change mechanisms and driving factors in estimating UHI.

Previous research on UHI mechanisms mostly focused on the relationships of UHI intensity with meteorological variables or city populations^5,17–19^. It did not fully explain the physical aspects of the estimated UHI. Recently, Lee *et al*.^20^ derived an equation using an energy balance system to analyze factors that contribute to the surface temperature contrast between land uses based on biophysical mechanisms. Zhao *et al*.^21^ modified a method from Lee *et al*.^20^ to analyze the driving factors of the UHI phenomenon in the US, which included incorporating aspects of storage change and anthropogenic heat into the original equation. However, there are few studies related to the influence of city landscapes on the pattern and magnitude of the driving factors that cause UHI based on biophysical mechanisms. In addition, the impact of urbanization on biophysical mechanisms during natural phenomena such as the El Niño-Southern Oscillation (ENSO) is not fully understood, and few studies have been conducted on this issue.

**Figure 1:**
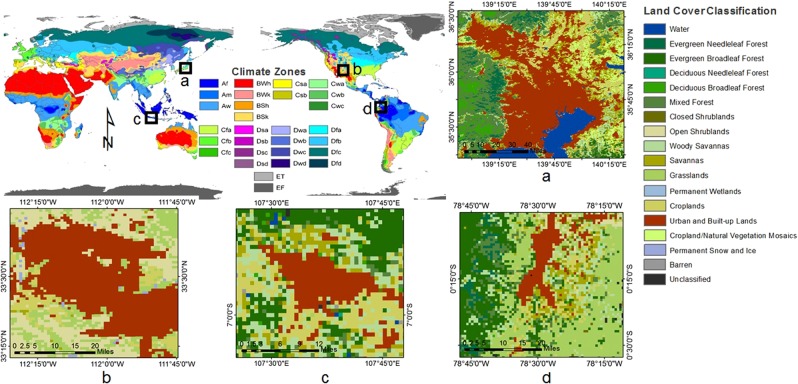
Geographical overview of the study sites including the climate and land cover types of (**a**) Tokyo, (**b**) Phoenix, (**c**) Bandung, and (**d**) Quito sites. The climate types were based on Köppen–Geiger classification (Peel *et al*.^50^), and the land cover classification was based on the IGBP land classification system.

In this study, we utilized the CLM to quantify UHI intensity in the cities of Tokyo, Phoenix, Bandung, and Quito, with different urban landscape structures and climate zones (Fig. 1, Table 1). First, we analyzed the contributions of the major biophysical drivers to the magnitude of UHI intensity based on the first derivation of linearized energy balance and compared this to the derived UHI intensity from Moderate Resolution Imaging Spectroradiometer (MODIS) LST products. Biophysical mechanism analysis of other important factors related to urbanization, such as city landscape, was also carried out. Lastly, variants in the driving factors and intensity of the UHI were analyzed during ENSO events that occurred in those cities.

**Table 1:** Overview of geographical, morphological, and climate characteristics describing the selected cities.

No.	City Name	Country	Height to Width Ratio (H/W)	Building Height (m)	Latitude	Longitude	Climate Type	Annual Precipitation (mm)	Annual Temperature (°C)	Elevation (m)
1	Tokyo	Japan	1.8	45	35.68° N	139.68° E	Humid subtropical	1,530	15.4	40
2	Phoenix	US	0.5	12	33.45° N	112.07° W	Hot desert	201	24.0	331
3	Bandung	Indonesia	0.7	13	6.90° S	107.60° E	Tropical monsoon	1,838	23.2	768
4	Quito	Ecuador	1.6	40	0.23° S	78.52° W	Subtropical highland	1,098	15.6	2,850

The morphological characteristics based on the default urban surface dataset were developed by Jackson *et al*.^29^, while the climate type was based on Köppen-Geiger classification (Peel *et al*.^50^).

## Results and Discussions

### Model evaluations

UHI intensity exhibited different characteristics during the daytime and nighttime, and its calculations depended on the ability of the CLM to simulate LST especially for urban surfaces provided by the CLMU. The ability of the CLMU to estimate LST was demonstrated by the high degree of agreement between simulated and observed temperatures from the flux tower for daytime and nighttime, with correlation coefficient (R) and index of agreement (IOA) values higher than 0.85. The CLMU produced a low error value with bias and root mean square error (RMSE) values lower than 5 K (Table 2).

**Table 2:** Statistical analysis of observed and simulated land surface temperature (LST) from the flux tower, CLM using GLDAS and Qian data at the Tokyo site, and CLM using GLDAS data at the Phoenix site in the daytime and nighttime with unit K for bias and root mean square error (RMSE).

	LST_Daytime_			LST_Nighttime_		
	Tokyo		Phoenix	Tokyo		Phoenix
	GLDAS	Qian	GLDAS	GLDAS	Qian	GLDAS
Bias	−0.91	2.02	−0.60	1.53	1.38	1.15
RMSE	1.87	4.13	2.87	2.15	3.87	2.42
IOA	0.99	0.92	0.97	0.98	0.93	0.98
R	0.98	0.89	0.95	0.98	0.88	0.97

**Figure 2:**
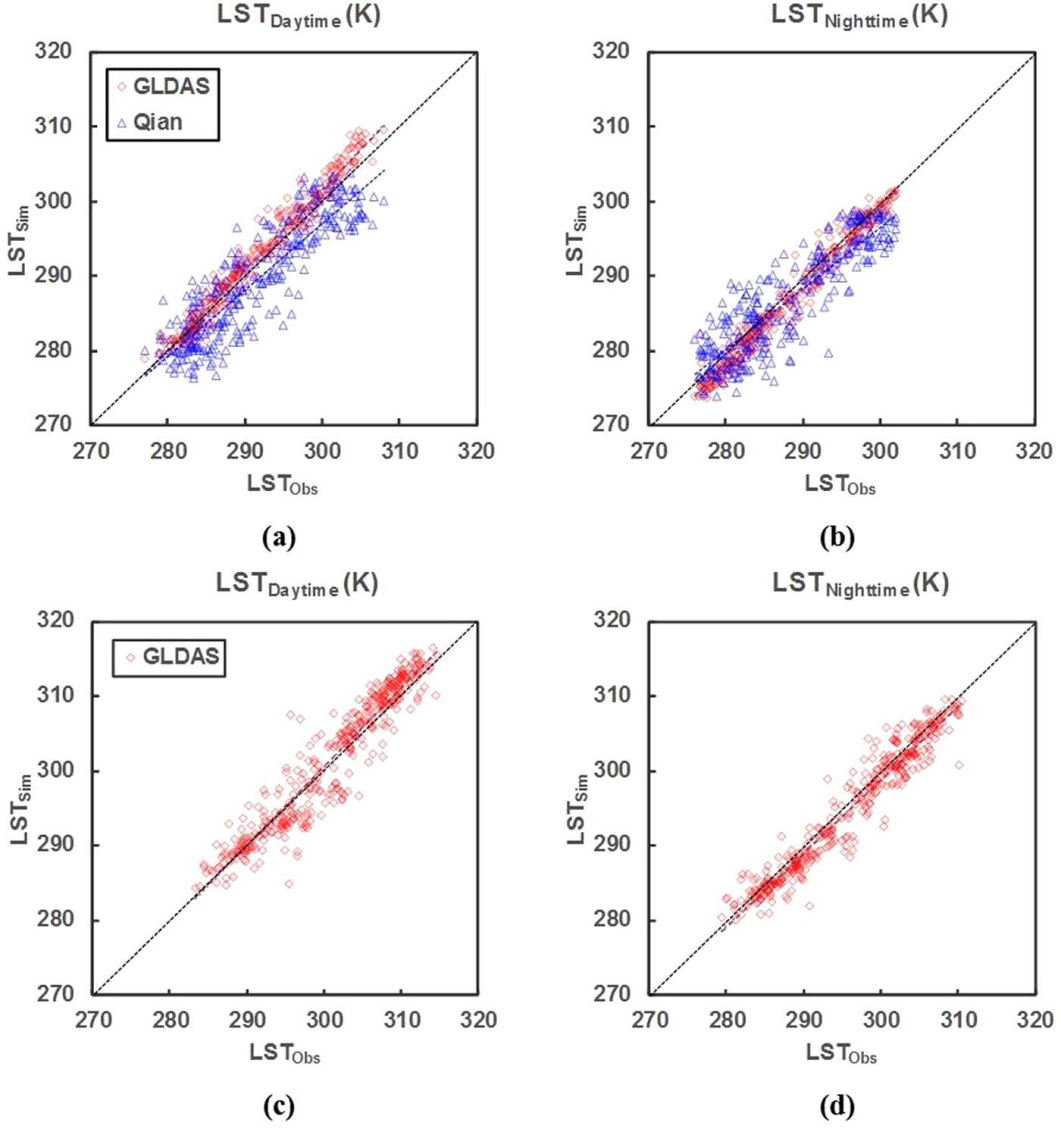
Comparisons of land surface temperature (LST) between observation (Obs) of flux towers and simulation (Sim) using the CLM with GLDAS and Qian data during the (**a**) daytime and **(b**) nighttime at the Tokyo site from April 1, 2001, to May 31, 2002, and during (**c**) daytime and (**d**) nighttime at the Phoenix site from January 1, 2012, to December 31, 2012.

CLM outputs using different atmospheric forcing data such as Global Land Data Assimilation System (GLDAS) and Qian data were compared with the Kugahara flux tower to assess the sensitivity of different forcing data, which could influence biophysical driver calculations. The times of 13:00 and 01:00, which were similar to the MODIS overpass time, were selected representing LST during the daytime and nighttime to avoid the effect of the diel cycle. The LST values at the selected times were used in UHI calculations from the model. Figure 2a,b compare the LST of CLM using different forcing data from the Kugahara flux tower during daytime and nighttime. Both forcing datasets correlated well with observations during daytime and nighttime with a slight bias (Fig. 2a,b). GLDAS data improved the CLM output more than Qian data, as shown by a higher R value from CLM using GLDAS data than Qian forcing during the daytime (from 0.89 to 0.98) and nighttime (from 0.88 to 0.98) (Table 2). The estimated LST using GLDAS produced biases lower than 2 K with slight overestimation during the daytime and underestimation during the nighttime (Table 2). This result was similar to that of a previous study by Meng^15^, who used GLDAS data in IUM and CoLM. This may have occurred because the GLDAS outperformed Qian data by supplying higher spatial and temporal resolution that determines the accuracy of the forcing variables, especially precipitation, which is one of the most important variables for hourly output from the CLM^22^.

The simulated LST at the Phoenix site using GLDAS showed good agreement with the observed data, with an R value of 0.95 (0.97) and an IOA value of 0.97 (0.98) for daytime (nighttime) (Table 2). The results had a low error where the bias and RMSE values lower than 3 K. Similar to the Tokyo site, estimation at the Phoenix site captured the LST reasonably well with slight overestimation during the daytime (Fig. 2c) and underestimation during the nighttime (Fig. 2d). This condition could be due to the limited urban parameterization of the model. For example, the anthropogenic heat flux in recent models only includes conventional variables that can underestimate the temperature at night^21,23,24^.

The UHI based on biophysical mechanism analysis depends on simulated energy fluxes, such as sensible heat flux (*Q*_H_) and latent heat flux (*Q*_E_), especially for the second and third terms of Eq. (1). The second term was affected by the energy redistribution factor associated with roughness change (∆*f*_1_) from Eq. (4), which used the estimated aerodynamic resistance (*r*_a_) using *Q*_H_ from CLM (Eq. 6) because *r*_a_ values were not provided directly by the CLM. The third term represented the influence of the Bowen ratio difference, which depended on both the simulated *Q*_E_ and *Q*_H_. The CLM estimation of *Q*_H_ showed a strong similarity to observed data for both Tokyo and Phoenix sites, with R and IOA values above 0.5, while the *Q*_E_ estimation showed moderate agreement, with R and IOA values above 0.4 (Table 3). Based on the statistical results shown in Table 3, the estimation results of CLM were considered reliable for use in UHI attribution analysis.

**Table 3:** Statistical analysis of observed and simulated sensible heat flux (Q_H_) and latent heat flux (Q_E_) from the flux tower, CLM using Qian and GLDAS at the Tokyo site, and CLM using GLDAS at the Phoenix site with bias and RMSE in W m^−2^.

	*Q*_H_			*Q*_E_		
	Tokyo		Phoenix	Tokyo		Phoenix
	GLDAS	Qian	GLDAS	GLDAS	Qian	GLDAS
Bias	−6.75	−10.47	7.65	−7.81	−6.55	−21.35
RMSE	47.00	58.58	28.09	34.82	39.47	33.18
IOA	0.84	0.75	0.93	0.76	0.68	0.41
R	0.73	0.51	0.89	0.61	0.49	0.45

We compared the estimation of energy fluxes using different forcings with available data from the flux tower in Tokyo to show the sensitivity output using well-known atmospheric data. The use of the GLDAS forcing produced notable improvement in the R of *Q*_H_ (*Q*_E_) from 0.51 to 0.73 (from 0.49 to 0.61) compared with the Qian forcing and reduced bias to approximately 3.72 (1.26) W m^−2^ and RMSE to approximately 11.58 (4.65) W m^−2^ (Table 3) for *Q*_H_ (*Q*_E_).

**Figure 3:**
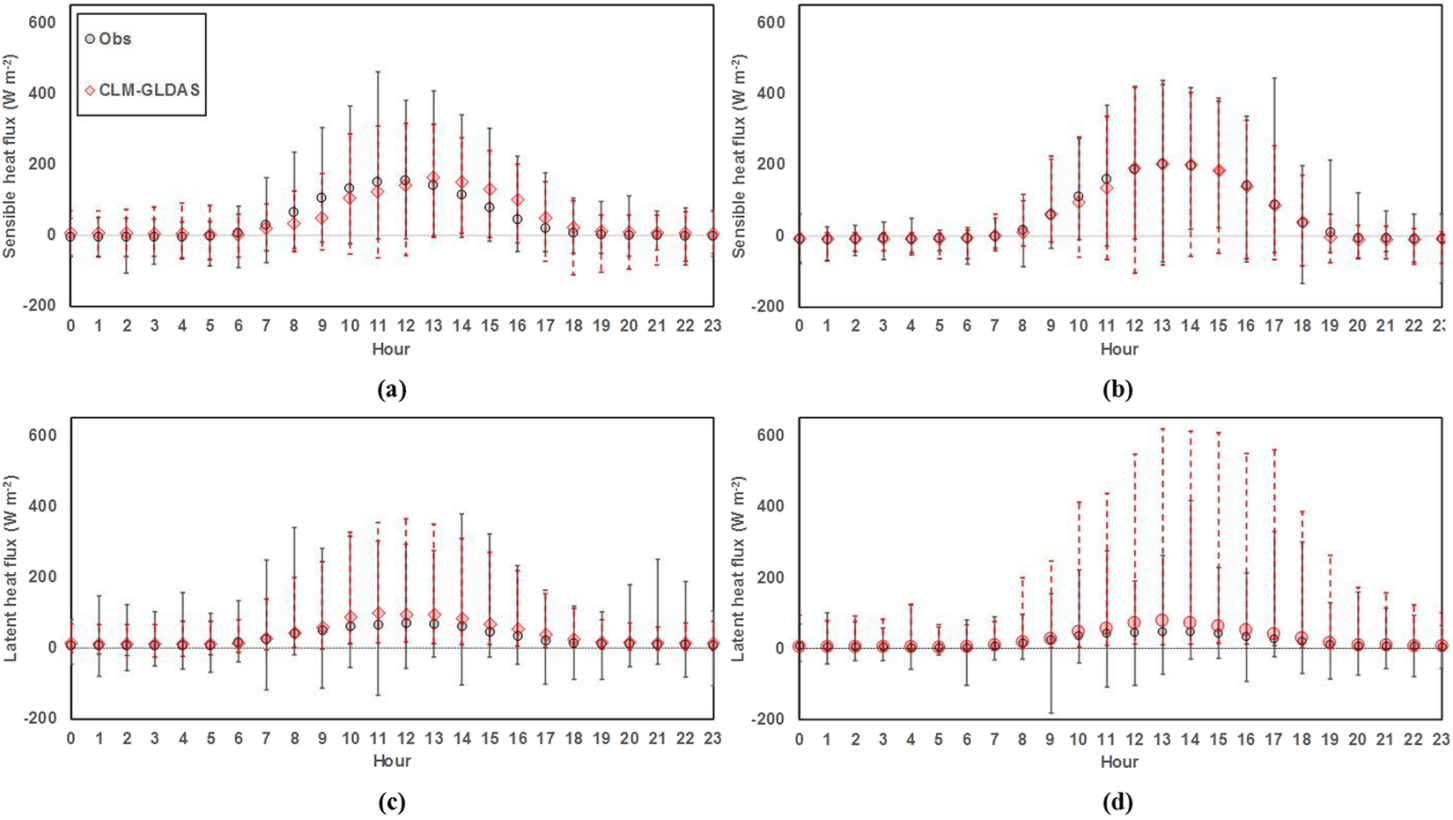
Observed and simulated average diurnal cycles of sensible heat flux (Q_H_) and latent heat flux (Q_E_) using GLDAS at the Tokyo (**a,c**) and Phoenix (**b**,**d**) sites. Error bars represent the range of average value to maximum and minimum values over the specific hour.

The model could depict the average diurnal pattern compared to observation data (Fig. 3). At the Tokyo site, the model slightly underestimated *Q*_H_ from 7 am until the middle of the day (12 pm) and then overestimated until 7 pm (Fig. 3a), while *Q*_E_ exhibited a small overestimation during the daytime from 10 am until 6 pm (Fig. 3c). The value of *Q*_E_ decreased over time, and more energy was converted to *Q*_H_ after midday, which resulted in overestimation because of the limited water capacity of roofs and pervious surfaces^25^. In the morning, the slight overestimation of *Q*_E_ (Fig. 3c) was caused by evaporation of adequate water on the surface, resulting in slight underestimation of *Q*_H_ (Fig. 3a). On the other hand, the simulated *Q*_H_ at the Phoenix site well depicted the pattern of observed data with a slight overestimation from 10 am to 11 am (Fig. 3b), while the simulated *Q*_E_ showed a similar diurnal pattern to that of the observed *Q*_E_ with only a slight overestimation during the daytime (Fig. 3d). The *Q*_E_ parameterization on the urban surface, which depended largely on roof and pervious surface evaporation, generated rapid evaporation from the model, especially roof evaporation during high sunlight radiation hours^25^. Overall, the CLM followed the pattern of observed data with slight difference at the Tokyo and Phoenix sites.

### Biophysical drivers of UHI

We analyzed the relationship between estimated UHI from 15-year outputs of CLM and biophysical drivers of surface albedo change (E1), convection reduction (E2), evaporation reduction (E3), heat storage change (E4), and anthropogenic release (E5) for daytime and nighttime. We also identified the influence of local climate background and urban canyon morphology on UHI intensity in Tokyo, Phoenix, Bandung, and Quito, as well as the effect of latitude region, which determined the amount of solar radiation received by the surfaces.

#### Local climate effects on biophysical mechanism findings

**Figure 4:**
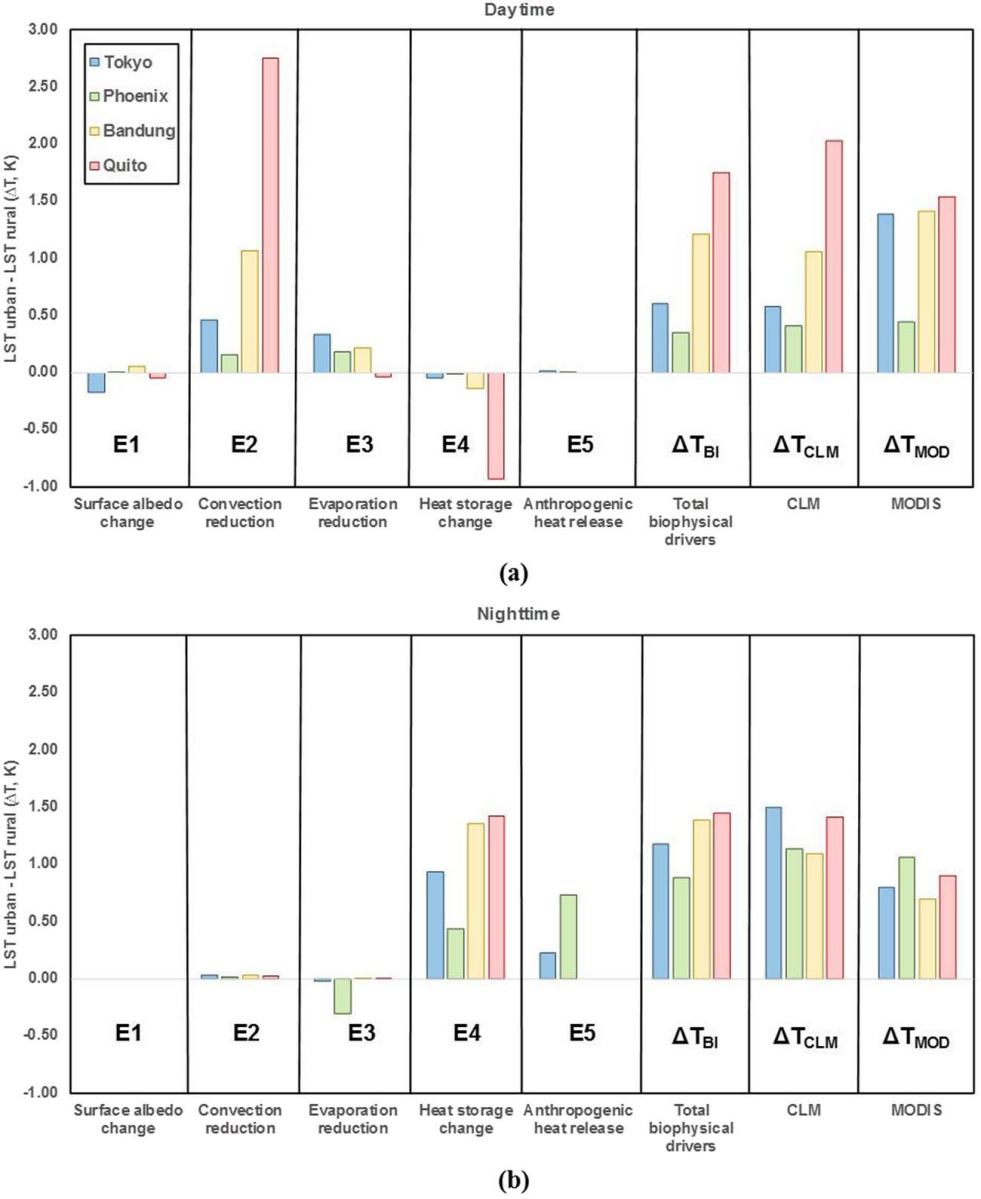
Estimation of UHI during (**a**) daytime and (**b**) nighttime from biophysical drivers of surface albedo change (E1), convection reduction (E2), evaporation reduction (E3), heat storage change (E4), and anthropogenic release (E5); total biophysical drivers (Δ*T*_BI_) from E1 to E5; CLM (Δ*T*_CLM_); and MODIS (Δ*T*_MOD_) at the Tokyo, Phoenix, Bandung, and Quito sites. E1 to E5 represent terms 1 to 5 of Eq. (1), respectively.

The analysis of UHI based on the linearized energy balance equation showed different patterns during daytime and nighttime (Fig. 4). We found that the reductions in convection (E2) and evaporation (E3) for urban areas contributed significantly to UHI intensity during daytime, while biophysical drivers of surface albedo change (E1), heat storage change (E4), and anthropogenic release (E5) had weaker influence on the UHI intensity.

During the daytime, the more influential driver of the five biophysical drivers on UHI intensity was convection reduction (E2 in Fig. 4a). Bandung and Quito, which have tropical climates, experienced less efficient convection due to the higher aerodynamic resistance of urban areas in comparison with rural areas, which enhanced the UHI intensity and reduced the capability to dissipate from urban to rural areas (E2 in Fig. 4a)^21^. Similarly, Tokyo, which has a humid climate, was affected mainly by convection associated with aerodynamic roughness (E2 in Fig. 4a). However, in arid Phoenix, the UHI was influenced strongly by the difference in evaporation processes between urban and rural areas because of its limited water surfaces, which reduced the amount of evaporation in the urban area compared to rural areas (E3 in Fig. 4a). This led to a higher LST for urban areas and increased UHI intensity^21,24^.

Tokyo and Phoenix (also Bandung and Quito), which are at the same latitude, showed a distinct pattern of convection reduction in the daytime (Table 1, E2 in Fig. 4a). Phoenix experienced more efficient convection (as indicated by a lower *∆T*) than that of Tokyo during the daytime (E2 in Fig. 4a). This might be due to the different compositions of vegetation between the Phoenix and Tokyo grids in the model. Phoenix vegetation was shorter in the rural side due to the desert ecosystem^21^, while Tokyo had tall and dense vegetation^26^. As described in the surface dataset used in the model, the rural area of Tokyo consisted mostly of temperate needleleaf evergreen trees (NET) and broadleaf evergreen trees (BET) with canopy top (bottom) height, *Z*_top_ (*Z*_bot_), of 17 m (8.5 m) and 20 m (11.5 m), respectively (Table 2.2 in Oleson *et al*.^27^). This vegetation structure in Tokyo was considered dense (Zhao *et al*.^21^) and could give rise to higher aerodynamic roughness of the surrounding rural area compared to the urban area enhancing dissipation of sensible heat from rural areas in comparison with urban areas and leading to a warmer urban LST. Similarly, tropical city (Bandung and Quito) vegetation comprised mainly BET–tropical with *Z*_top_ and *Z*_bot_ values of 30 m and 1 m (Table 2.2 in Oleson *et al*.^27^), respectively, which resulted in higher roughness in rural areas and led to greater convection reduction in both Bandung and Quito (E2 in Fig. 4a). Based on a comparison of convection for these cities, convection in Bandung was more efficient than in Quito (E2 in Fig. 4a). Despite similar neighborhood landscapes with mountains and location near the equator, the elevation difference of Bandung and Quito could have affected the difference in convection efficiency. Quito, which is at a higher elevation than Bandung, potentially received more solar radiation, which contributed to heat dissipation from the urban compared to the rural area (Table 1). As a result, more incoming radiation in Quito would be trapped inside the urban canyon, which led to a higher LST in this area.

During the nighttime, changes in storage change (E4) and anthropogenic heat release (E5) were the major contributing biophysical drivers compared with surface albedo change (E1), convection reduction (E2), and evaporation reduction (E3) (Fig. 4b). In Tokyo, Bandung, and Quito, heat storage release had a greater impact on UHI intensity (E4 in Fig. 4b) because the urban structure and characteristics of impervious surfaces with a higher thermal admittance meant that urban areas emitted heat faster than rural areas comprising vegetation and bare land^25^. The impervious surfaces stored more heat than rural areas during the daytime, increasing the heat released at nighttime. In contrast, anthropogenic heat release was the dominant biophysical driver in Phoenix due to greater usage of heating and air conditioning^28^ (E5 in Fig. 4b). Phoenix, which has a hot arid desert climate with a long and extremely hot summer, requires ample air conditioning to reduce indoor temperatures.

With regard to location, low latitude tropical cities such Bandung and Quito had higher UHI intensity caused by storage heat change (E4) in the nighttime than did subtropical cities such as Tokyo and Phoenix, due to different amounts of solar radiation received throughout the year. The effects of anthropogenic heat (E5) did not greatly affect the UHI intensity at nighttime in the tropical cities compared with the subtropical cities. Heating systems, which were the major source of anthropogenic heat in the CLM, are not typically used in tropical areas because these areas have only two seasons (dry and wet), unlike temperate regions with a winter season. The CLM estimated anthropogenic heat flux using the default urban surface dataset, in which the tropical area had lower energy requirements for maintaining temperature of the building^29^.

In general, *∆T*_BI_ results showed a similar pattern to *∆T*_CLM_ among sites, as *∆T*_CLM_ was calculated directly using the LST of CLM for all sites at daytime and nighttime (Fig. 4). *∆T*_MOD_ also showed good agreement with UHI estimation including *∆T*_BI_ and *∆T*_CLM_. The differences in *∆T* magnitude between total biophysical driver calculation and CLM caused by Eq. (1) considering only the major drivers in biophysical mechanism calculation. On the other hand, the methodology for calculation of anthropogenic flux in the model considered only primitive anthropogenic schemes such as fluxes of heating and air conditioning, waste heat of heating and air conditioning, and the heat removed by air conditioning, and neglected the flux from urban traffic^21,24^. This resulted in underestimation of UHI intensity, especially at nighttime. Small variations during the night were a result of biogeochemical effects of aerosols^23^. The results of this study showed a higher UHI intensity during the nighttime except in Quito. In this case, precipitation could explain the higher UHI effect during the daytime because it greatly affected evapotranspiration. Li *et al*.^30^ concluded that local background precipitation strongly controls rural temperatures. Quito’s high elevation and wet climate (high precipitation) resulted in a lower rural temperature and a high UHI intensity during the daytime. Similar findings were reported by Zhao *et al*.^21^ and Cao *et al*.^23^, where humid regions had larger UHI intensity during daytime as denser vegetation in the rural surface produced higher evapotranspiration rates than did the urban surface.

#### The influence of urban landscape on the biophysical mechanism

The influences of urban canyon structure on UHI intensity from each biophysical driver were analyzed using urban morphology indices such as the ratio of the height to width (H/W) of the urban canyon and the fractions of the roof, pervious, and impervious surfaces in cities at the same latitude. The morphology of the urban canyon was considered to have a significant effect on UHI intensity because it determined the absorption and reflection of solar radiation and the heat storage capacity of urban surfaces^1,31^.

During the daytime, urban morphology affected the magnitude of biophysical drivers of surface albedo change (E1), convection reduction (E2), evaporation reduction (E3), and storage heat change (E4), while anthropogenic heat (E5) was less impacted. The cooling effect with negative *∆T* in surface albedo change (E1) related to the radiation effect in Tokyo and Quito, which could be influenced by the higher roof fractions in Tokyo (0.7) and Quito (0.65) compared with Phoenix (0.55) and Bandung (0.35) (E1 in Fig. 4a). The high roof fraction in the city could reflect a large portion of solar radiation and reduce the absorption of solar radiation by urban surfaces^1^.

The ability of the convection process (E2) to efficiently dissipate heat from each surface in urban and rural areas was influenced by the aerodynamic roughness of the area^21^. The comparison of standalone urban canyon geometry of the cities could not determine the UHI intensities due to a complicated relationship between urban geometry and aerodynamic resistance^30^. However, UHI intensity was contributed to by both urban and rural landscapes, which exhibited differences in aerodynamic resistance (*∆r*_a_) (Eqs. (1) and (4)) and convection. Figure 4a shows that Tokyo (Quito) had a larger *∆T* for convection reduction compared to Phoenix (Bandung) due to a higher *∆r*_a_ between urban and rural areas. As a result, UHI intensity in Tokyo (Quito) was higher than that in Phoenix (Bandung) during the daytime (Fig. 4a). The rural surface with dense vegetation in Tokyo generated lower aerodynamic resistance than that in Phoenix, causing a higher *∆r*_a_ between urban and rural surfaces in Tokyo than in Phoenix, which had small stature vegetation on the rural surface. Similarly, based on the surface dataset, Quito had denser vegetation that resulted in greater *∆r*_a_ than Bandung.

The urban morphology of Tokyo with high H/W reduced evaporation (E3) and resulted in a higher *∆T* during the daytime compared with Phoenix (E3 in Fig. 4a). This was caused by the narrow sky view factor of urban canyon that limited the space for incoming radiation to the middle part of the urban canyon, which greatly affected evaporation. Despite a high H/W, Quito showed a slightly negative *∆T* (E3 in Fig. 4a) due to its high elevation (2,850 m in Table 1) and location near the equator, resulting in a high intensity of radiation to support evaporation.

City morphology significantly affected heat storage change in the daytime (E4 in Fig. 4a). The heat stored in the surface reduced the LST due to the decreased heat flux on the surface. Tokyo (Quito) with higher H/W and roof fraction stored more heat during the daytime than did Phoenix (Bandung), indicated by the negative *∆T* values where urban areas stored more heat than rural surfaces (E4 in Fig. 4a). Tropical cities of Bandung and Quito received more solar radiation compared to subtropical areas of Tokyo and Phoenix. Tropical cities therefore stored more solar radiation in the daytime and further decreased the UHI effect, producing larger differences in LST compared to subtropical cities.

During the nighttime, the urban structure influenced evaporation reduction (E3) and storage heat change (E4) more than other biophysical drivers. The evaporation reduction aspect in Tokyo and Phoenix showed a cooling effect with negative *∆T* (E3 in Fig. 4b). Nighttime evaporation occurred mainly through the soil because there was no source of light to support transpiration and took place primarily in the urban area. The advective effect could explain enhanced evaporation from pervious surfaces (bare soil) in urban area^32^. The differences in temperature and humidity between pervious (bare soil) and built surfaces were the source of warm-air advection to pervious surfaces, enhancing evaporation^32^. However, the rural surface consisting largely of vegetation had homogeneous temperatures among the functional types of plants, providing no source of heat for evaporation at nighttime. Because the urban area of Tokyo had a lower pervious fraction than that of Phoenix based on the CLM surface dataset, the amount of bare soil in Tokyo was lower. As a result, there was more significant soil evaporation in Phoenix, and the city cooled off more at nighttime than did Tokyo. Despite the different types of vegetation in rural areas of Tokyo and Phoenix, the contribution to evaporation at nighttime was not significant, as the transpiration process in vegetation depends on solar radiation.

The heat storage change aspect (E4) in Tokyo (with higher H/W and roof fraction) was associated with a higher *∆T* value compared to that of Phoenix (with lower H/W and roof fraction), despite their similar latitudes (E4 in Fig. 4b). Likewise, Quito with higher H/W and roof fraction and Bandung with lower H/W and roof fraction showed similar trends in UHI intensity. Cities with higher H/W values stored large amounts of radiation on the urban surface in the daytime, which resulted in greater heat release at nighttime.

Overall, cities with a higher H/W such as Tokyo (Quito) had a higher UHI intensity than those with lower H/W such as Phoenix (Bandung) based on both *∆T*_BI_ and *∆T*_CLM_ for daytime and nighttime (Fig. 4). As a comparison, *∆T*_MOD_ showed a similar pattern to *∆T*_BI_ and *∆T*_CLM_ in daytime (Fig. 4a), and cities with higher H/W had a larger UHI at the same latitude. However, *∆T*_MOD_ showed a lower *∆T* value compared to that from *∆T*_BI_ and *∆T*_CLM_, especially in Tokyo at nighttime (Fig. 4b). The UHI intensity from MODIS was calculated from the urban cores with a small vegetation fraction, while the urban surface in the climate model had no vegetation, which affected the amount of heat released from the surfaces^21^.

### Characteristics of UHI during the ENSO period

**Figure 5:**
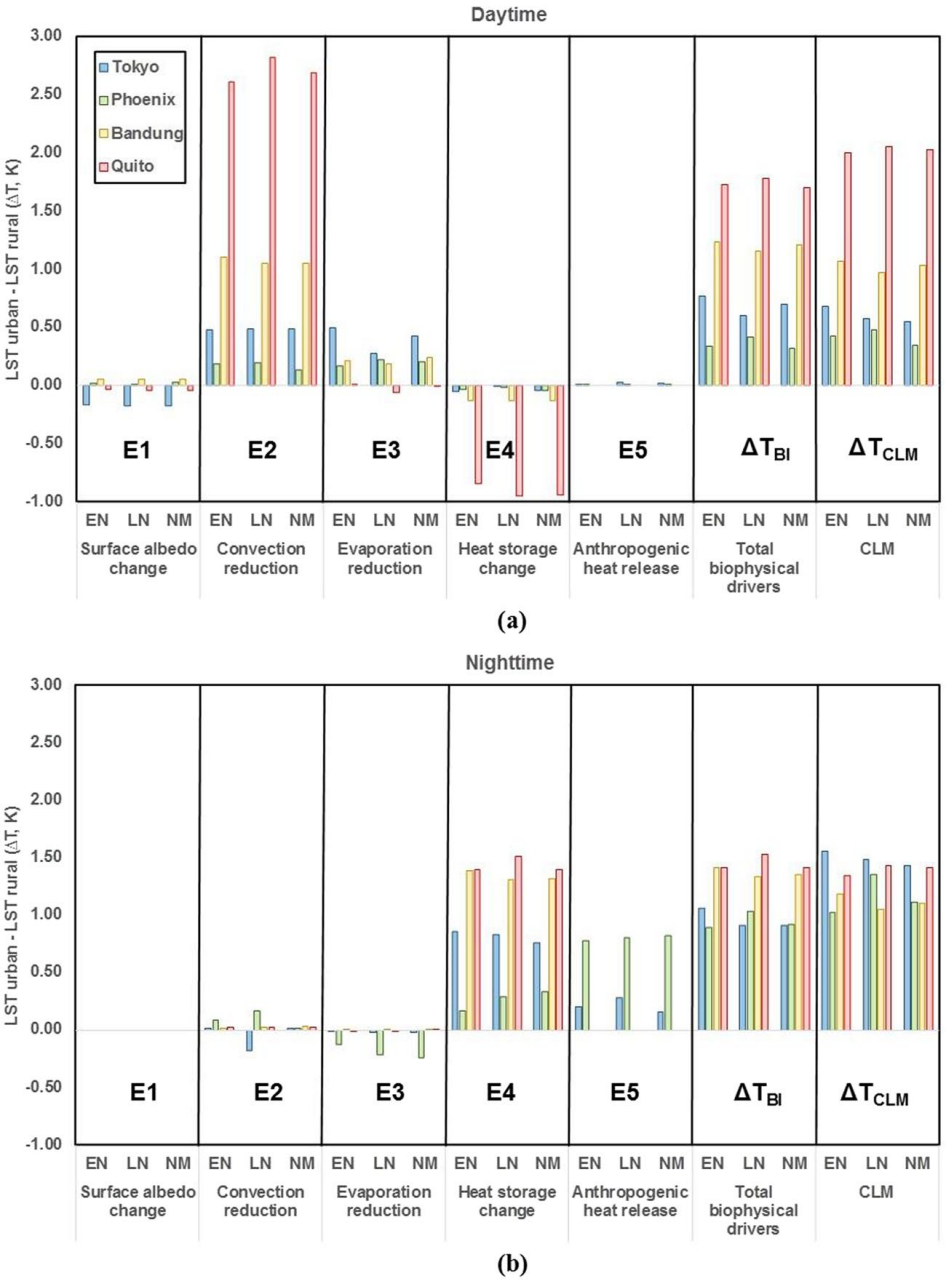
Estimation of UHI during (**a**) daytime and (**b**) nighttime from biophysical drivers of surface albedo change (E1), convection reduction (E2), evaporation reduction (E3), heat storage change (E4), and anthropogenic release (E5); total biophysical drivers (ΔT_BI_) from E1 to E5, and the CLM (ΔT_CLM_) under El Niño (EN), La Niña (LN), and Normal (NM) conditions at the Tokyo, Phoenix, Bandung, and Quito sites.

Figure 5 shows the different trends of each ENSO event for biophysical drivers and the UHI intensity from total biophysical drivers and CLM, and Table 4 presents the differences of UHI intensities between El Niño and La Niña events (*∆T*_EL-LN_). The result showed that ENSO events influenced mainly UHI intensity through convection reduction (E2) and evaporation reduction (E3) during the daytime. At nighttime, convection reduction (E2) and storage heat change (E4) were affected (Table 4).

**Table 4:** Differences in Δ*T* (K) during ENSO events of El Niño (EN) and La Niña (LN) in daytime and nighttime at Tokyo, Phoenix, Bandung, and Quito sites based on biophysical drivers of surface albedo change (E1), convection reduction (E2), evaporation reduction (E3), heat storage change (E4), and anthropogenic release (E5); total biophysical intrinsic drivers (ΔT_BI_) from E1 to E5; and CLM (ΔT_CLM_).

	Δ*T*_EL-LN_ (Daytime)				Δ*T*_EL-LN_ (Nighttime)			
	Tokyo	Phoenix	Bandung	Quito	Tokyo	Phoenix	Bandung	Quito
Surface albedo change (E1)	0.01	0.01	0.00	0.00	0.00	0.00	0.00	0.00
Convection reduction (E2)	−0.01	−0.01	0.05	−0.22	0.19	−0.09	−0.01	0.00
Evaporation reduction (E3)	0.23	−0.06	0.03	0.06	0.01	0.09	0.01	0.00
Heat storage change (E4)	−0.04	−0.02	0.00	0.10	0.02	−0.13	0.08	−0.11
Anthropogenic heat release (E5)	−0.02	0.00	0.00	0.00	−0.08	−0.03	0.00	0.00
Total biophysical drivers (Δ*T*_BI_)	0.16	−0.08	0.08	−0.05	0.15	−0.16	0.08	−0.11
CLM (Δ*T*_CLM_)	0.11	−0.06	0.10	−0.06	0.07	−0.33	0.14	−0.09

In daytime analysis, the largest difference during ENSO events was shown for *∆T* from evaporation reduction driver (E3) at Tokyo, which is at a high latitude (E3 in Fig. 5a, Table 4). The different patterns of evaporation reduction between Tokyo (0.23 K) and Phoenix (−0.06 K) during ENSO events at the same latitude are presented (Table 4). In Tokyo, the maximum UHI related to evaporation reduction occurred during El Niño, while Phoenix reached the peak during La Niña (E3 in Fig. 5a) due to the lower precipitation associated with La Niña conditions in Southern America, which enhanced the LST of surfaces^33^. Due to the limited water available to evaporate caused by low precipitation, the vegetation fraction played a more important part in water storage for evaporation compared with bare soil. As a result, the rate of evaporation in rural areas, which comprised water retaining in roots and leaves of vegetation, was higher than that in urban areas. In contrast, the rate of evaporation in urban area was relatively lower because the pervious surfaces in this area, which consisted primarily of bare soil, did not have any vegetation to store water. In Phoenix, the high rate of reduced evaporation in urban compared to rural due to fewer precipitation events resulted in a higher UHI during La Niña. A similar process occurred in Tokyo where precipitation was lower during El Niño. However, no significant difference in evaporation reduction at lower latitude cities of Bandung and Quito was seen between ENSO events. Adequate water on the surface due to the humid tropical climate and the solar radiation received during the summer months support evaporation from pervious surfaces in urban land units, especially bare soil. In contrast, convection reduction (E2) had a major impact on increased UHI intensity during ENSO events in Bandung and Quito (E2 in Fig. 5a). The decreasing convection during La Niña (El Niño) in Quito (Bandung) resulted in a 0.22 K (0.05 K) higher UHI intensity compared to that during El Niño (La Niña) event (Table 4, E2 in Fig. 5a). The ocean circulation anomaly due to ENSO events caused a difference in local effects between the western and eastern areas of the Pacific Ocean. A higher sea surface temperature (SST) in the eastern part during El Niño enhanced convection and resulted in more precipitation, while the inverse process occurred in western areas^34^.

At nighttime, ENSO events affected convection reduction (E2) more in subtropical cities (Tokyo and Phoenix), especially in Tokyo, than in tropical cities (Bandung and Quito) (Table 4). La Niña condition caused higher SST near Tokyo Bay than under normal conditions, which enhanced the convection process in the urban and rural areas and resulted in lower UHI in comparison to El Niño^34^. The high precipitation and cloud cover fraction in Tokyo Bay produced lower LSTs under La Niña condition in comparison with under the El Niño period, which influenced the UHI intensity difference by 0.19 K (Table 4, E2 in Fig. 5b). La Niña condition decreased the LSTs of urban and rural, while atmospheric temperature increased in comparison to that of the normal condition. Under a normal condition, the LST of urban was higher than the atmospheric temperature, and the LST of rural was lower than the atmospheric temperature. Absorption of heat by impervious surfaces was another reason for higher LST of urban during nighttime. Thus, during La Niña in Tokyo, the *r*_a_ in urban was lower than that in rural because its calculation was influenced by the difference between *T* and *T*_a_ (Eq. 6) and produced a result with a lower UHI compared to that of El Niño. Despite being located at the same latitude, Phoenix experienced a higher UHI during La Niña due to lower convection efficiency compared with other phases of ENSO (E2 in Fig. 5b). The lower SST near the southern U.S. (Phoenix) decreased convection^33^, which contributed to a reduction in heat dissipation in both urban and rural areas. The change in heat storage (E4) in Phoenix at nighttime produced a higher UHI intensity during La Niña in comparison with El Niño, while it was higher during El Niño in Tokyo (E4 in Fig. 5b). Similarly, Bandung had a higher UHI intensity caused by storage heat during El Niño, while Quito reached the maximum UHI intensity during La Niña (E4 in Fig. 5b). The variability in solar radiation during the daytime, influenced by ENSO events, determined the amount of heat stored by urban surfaces. Precipitation intensity and occurrence could be other causes of UHI differences in term of storage heat change at nighttime during ENSO events. For example, the temperature on rural surface was lower than that on the urban surface after several precipitation events, which affected the moisture content of soil and vegetation.

Overall, ENSO events enhanced UHI intensity in comparison to the normal state, as determined by local climate characteristics and urban morphology. Figure 5 shows that Tokyo and Bandung reached maximum UHI intensities for both *∆T*_BI_ and *∆T*_CLM_ under El Niño condition for both daytime and nighttime, while Phoenix and Quito had maximum UHI intensities during La Niña. Phoenix experienced the highest UHI difference between ENSO events compared to other cities, especially at nighttime, when it reached approximately 0.33 K (Table 4). This difference was supported by the hot arid climate of the area and was influenced by precipitation occurrence.

## Conclusions

In this study, we conducted an analysis of the deriving factors of UHI effect based on the mechanisms of biophysical drivers such as change in net radiation, aerodynamic resistance, Bowen ratio, storage heat, and anthropogenic release in cities with different characteristics. During the daytime, UHI intensity was affected primarily by the lower efficiency of the convection process in humid and tropical areas, while reduction of evaporation was the main issue in hot arid areas. However, the difference in storage heat between urban and rural surfaces mostly determined the UHI intensity at the nighttime in humid and tropical areas, while anthropogenic heat was the dominant factor in hot arid areas.

UHI intensity was also influenced by climate variability such as that caused by the ENSO. The different amounts of precipitation and convection due to ENSO events determined the magnitude of impact of each biophysical driver on UHI intensity, especially reduction of convection and evaporation for the daytime and convection reduction and storage heat change at nighttime. In the daytime, a lower efficiency of convection enhanced the UHI during El Niño events for western areas of the Pacific Ocean, such as Bandung and Tokyo, compared to La Niña events due to the colder SST in this area. Cities on the eastern side of the Pacific Ocean, such as Quito and Phoenix, experienced higher UHI intensity caused by a reduction in convection efficiency during the La Niña phase, because the SSTs were cold near those areas. The amount of precipitation during ENSO events greatly influenced the reduction of evaporation in the subtropical cities. These results showed that a city with a rough landscape, indicated by the H/W of the urban canyon, had a higher UHI than that of a city with lower roughness at the same latitude. For future study, development of a new urban surface dataset that includes urban fractions and morphology is required, as they are considered important factors in model outputs and UHI estimation. Furthermore, implementation of dynamic land use and combined use of socio-economic data when analyzing the effects of city planning, including inter-comparisons of land functional use within urban regions, can provide a wider perspective of urban development and the UHI phenomenon in mitigating heat stress.

## Methods

### Study area and datasets

As shown in Fig. 1, four study sites with different landscapes and climate characteristics were used in this study. Two study sites (Phoenix, Arizona, US, and Tokyo, Japan) with an urban flux tower were used to evaluate the characteristics of energy fluxes simulated by the CLM. The Tokyo site was also used to estimate the model sensitivity to different forcing data. The characteristics for each city are shown in Table 1.

The landscape of Tokyo consists of tall buildings with modern architecture and numerous parks and gardens^35^, while Phoenix has a relatively flat topography and is surrounded by mountains^36^. Furthermore, Bandung, which is one of the largest cities in Indonesia and is located on the equator, has a blend of architecture with many old buildings of Dutch architecture and native traditional architecture^37^. Quito, the capital city of Ecuador, has a city landscape that differs between modern high buildings in the north and industrial buildings in the south^38^.

#### Hydro-meteorological observation data

An urban flux tower was established at Maryvale in the city of Phoenix, (33.48° N, 112.14° W) with homogeneous building design (single-story, single-family, separated residences). The measurement was performed by the Central Arizona-Phoenix Long-Term Ecological Research. To avoid the influence of localized turbulence and material fluxes created by anomalous surface objects, the antenna was extended vertically approximately 22.86 m to sample neighborhood-scale fluxes. Several correction procedures were implemented to obtain high-quality data^39^. During processing of the flux tower data, noise was removed when the instantaneous values were ± 4σ, and the Webb–Pearman–Leuning (WPL)^40^ method was used to correct the density fluctuation effect of heat and water fluxes in flux tower data. Data from this site were provided from December 2011 to December 2012 at a 30-min resolution and were up-scaled to 1-hour for comparison to hourly outputs from the CLM. Chow *et al*.^39^ reported that the energy closure of this site was within the range of error calculated from several FLUXNET sites^41^.

The urban flux measurements were collected from May 2001 until April 2002 in Kugahara, Tokyo, Japan (35.57° N, 139.68° E). The instruments were installed in the backyard of a home at a height of 29 m. The residential neighborhood consists mainly of dense houses, paved roads, and small playgrounds. In general, the building height is 7.3 ± 1.3 m based on the average height of 500 buildings near the tower^26^. As with the Phoenix flux tower, fluxes at the Tokyo site were pre-processed, including implementation of WPL correction and coordinate rotation^26^. The data were estimated every 60 minutes using the eddy covariance (EC) method. We used only the available data without estimation for missing data due to sensor malfunction. The energy imbalance closure was smaller than typical (approximately 20%) due to systematic instrument installation, which is explained in a study by Moriwaki and Kanda^26^.

#### Satellite data

Remotely sensed data can be used to estimate the temperature difference between urban and rural regions^11^. The MODIS Aqua LST product (MYD11A2) used in this study has a 1 km spatial resolution for the study period from 2002 to 2014. This data provided 8-day LST observations with satellite overpass times around 13:30 and 1:30 local time. MYD11A2 is suitable for UHI detection because clouds were filtered out to avoid interference on surface temperature^13^. The atmospheric water vapor and haze effects were corrected using a generalized split windows algorithm with two longwave bands in the atmospheric windows^42^. The brightness temperature in MYD11A2 was corrected for the surface emissivity effect to produce the true LST^43^. We used MODIS land cover data (MCD12Q1) to delineate the urban pixels and exclude water and mountain pixels from MODIS LST data. The rural area was selected based on the rural pixels of the surrounding urban core following the method developed by Zhao *et al*.^21^. The UHI intensity was defined as the difference between LST from urban pixels and rural pixels. The UHI intensity derived from MODIS (*∆T*_MOD_) was presented along with the UHI intensity calculated from CLM (*∆T*_CLM_) for all cities.

### Model descriptions

The CLM, which is the land component of the Community Earth System Model (CESM), represents the interaction between land and atmosphere. The CLM is composed of five land units at the first hierarchy level: glacier, lakes, vegetation, wetland, and urban. The urban land unit is represented as several columns, such as roof, sunlit and shaded walls, and pervious and impervious canyon floors^1^, which is based on the canyon concept of Oke^44^. The complexity of urban surfaces is reduced to a single urban canyon consisting of a canyon floor, represented by width (W), and surrounded by two buildings, which are indicated by height (H) (Figure 1.3 in Oleson *et al*.^1^). The ratio of H to W (H/W) represents the morphological properties of the urban canyon^29^. The roof, sunlit wall, shaded wall, and impervious surface are non-functional hydrological columns in CLMU and are only able to intercept, store, and evaporate a limited amount of precipitation. On the other hand, pervious surfaces, such as residential lawns and parks, have active hydrological functions^45^.

The surface dataset of selected cities was constructed from the default surface dataset in CLM4, while parameterization for urban characteristics was developed by Jackson *et al*.^29^. In this study, we used these two forcing datasets, both widely known and used, to show the sensitivity of reanalysis atmospheric data on the differences in energy fluxes and LST. The model used the Qian reanalysis data and GLDAS data for forcings. The Qian reanalysis data are reconstruction climatology data from 1972 to 2004, which integrated US National Centers for Environmental Prediction and National Center for Atmospheric Research (NCEP-NCAR) reanalysis, observation-based analyses, and the observational record^46^, while the GLDAS is a comprehensive combination of ground and satellite observations^47^. The 3-hour data of all variables were used from GLDAS over a study period from 2000 to 2015. Meanwhile, the Qian data drew upon 1990 to 2004 records with the same time resolution of 3 hours for all variables except solar radiation and precipitation, which were only available as 6-hour data.

### UHI Offline calculation method

We used the method developed by Zhao *et al*.^21^ to analyze the UHI intensity in North American cities. CLM outputs were used to quantify the driver for the urban-rural temperature contrast based on biophysical mechanism, which included five major biophysical drivers: change in net radiation ($\Delta {R}_{n}^{\ast }$), aerodynamic resistance ($\Delta {r}_{a}$), Bowen ratio ($\Delta \beta$), storage heat ($\Delta {Q}_{s}$), and release of anthropogenic heat ($\Delta {Q}_{AH}$). The symbol $\Delta$ represents urban disturbance to rural and was calculated as urban flux minus rural flux for the specific variable. The total contributions of biophysical drivers to the surface temperature contrast (*∆T*_BI_) of urban to rural areas are expressed as follows:

1 $$\Delta {T}_{BI}=\frac{{\lambda }_{0}}{1+f}\Delta {R}_{n}^{\ast }+\frac{-{\lambda }_{0}}{{(1+f)}^{2}}({R}_{n}^{\ast }-{Q}_{s}+{Q}_{AH})\Delta {f}_{1}+\frac{-{\lambda }_{0}}{{(1+f)}^{2}}({R}_{n}^{\ast }-{Q}_{s}+{Q}_{AH})\Delta {f}_{2}+\frac{-{\lambda }_{0}}{1+f}\Delta {Q}_{s}+\frac{{\lambda }_{0}}{1+f}\Delta {Q}_{AH}$$

2 $$f=\frac{{\lambda }_{0}\rho {C}_{p}}{{r}_{a}}(1+\frac{1}{\beta })$$

3 $${R}_{n}^{\ast }=(1-a)K\downarrow +L\downarrow -(1-\varepsilon )L\downarrow -\varepsilon \sigma {T}_{a}^{4}$$

4 $$\Delta {f}_{1}=\frac{-{\lambda }_{0}\rho {C}_{p}}{{r}_{a}}(1+\frac{1}{\beta })\frac{\Delta {r}_{a}}{{r}_{a}}$$

5 $$\Delta {f}_{2}=\frac{-{\lambda }_{0}\rho {C}_{p}}{{r}_{a}}\frac{\Delta \beta }{{\beta }^{2}}$$

6 $${r}_{a}=\frac{\rho {C}_{p}(T-{T}_{a})}{{Q}_{H}}$$

where ${\lambda }_{0}$ is the sensitivity of the local climate (${\lambda }_{0}=1/4\varepsilon \sigma {T}^{3}$), $f$ is the energy redistribution factor, $a$ is the surface albedo, $K\downarrow$ is incoming solar radiation, $L\downarrow$ is incoming long-wave radiation, $\varepsilon$ is the surface emissivity, $\sigma$ is the Stefan–Boltzmann constant, ${R}_{n}^{\ast }$ is apparent net radiation, ${Q}_{s}$ is storage heat, ${Q}_{AH}$ is anthropogenic heat, $\varDelta {f}_{1}$ is the energy redistribution factor associated with roughness change, $\Delta {f}_{2}$ is the energy redistribution factor associated with Bowen ratio ($\beta$) change, ${r}_{a}$ is the aerodynamic resistance to dissipate sensible heat, $\rho$ is air density, ${C}_{P}$ is specific heat of air at constant pressure, $T$ is surface temperature, ${T}_{a}$ is air temperature at a reference height, and ${Q}_{H}$ is sensible heat flux.

The right-side of Eq. (1) represents surface albedo change (term 1, $\frac{{\lambda }_{0}}{1+f}\Delta {R}_{n}^{\ast }$), the change in aerodynamic resistance to dissipate heat or convection (term 2, $\frac{-{\lambda }_{0}}{{(1+f)}^{2}}({R}_{n}^{\ast }-{Q}_{s}+{Q}_{AH})\Delta {f}_{1}$), the reduction of evaporation (term 3, $\frac{-{\lambda }_{0}}{{(1+f)}^{2}}({R}_{n}^{\ast }-{Q}_{s}+{Q}_{AH})\varDelta {f}_{2}$), the change in storage heat (term 4, $\frac{-{\lambda }_{0}}{1+f}\Delta {Q}_{s}$), and the release of anthropogenic heat (term 5, $\frac{{\lambda }_{0}}{1+f}\Delta {Q}_{AH}$).

We calculated $\Delta T$ using the extracted variables from the model output at 13:00 and 01:00 for daytime and nighttime, respectively. These times were selected because the local surface temperatures potentially reach its maximum and minimum values at these respective times, and they are near the overpass time of data acquisition from MODIS Aqua^21^.

### El Niño–southern oscillation (ENSO)

ENSO is a climate variation observed as a periodic fluctuation of SST and atmospheric air pressure across the Pacific Ocean. There are three phases of ENSO: El Niño, La Niña, and Neutral or Normal, based on whether the state of the SST is above, below, or at normal conditions for the eastern part of the Pacific Ocean, respectively^48^. An SST above normal enhances convective activity, and vice versa.

To separate the analysis based on ENSO events, we used a well-known and widely used index, the Southern Oscillation Index^48^, which can be obtained from the Australia Bureau of Meteorology, to extract the CLM results during ENSO events. This index measures the difference in observed mean sea level pressure between Tahiti and Darwin, with a zero value as the average index, and is presented in standard deviation units^49^. An El Niño (La Niña) event is categorized by sustained negative (positive) values lower (higher) than −7 (+7).
